# De novo post-transplant thrombotic microangiopathy localized only to the graft in autosomal dominant polycystic kidney disease with thrombophilia

**DOI:** 10.12861/jrip.2015.28

**Published:** 2015-11-30

**Authors:** Davide Rolla, Iris Fontana, Jean Louis Ravetti, Luigina Marsano, Diego Bellino, Laura Panaro, Francesca Ansaldo, Lisa Mathiasen, Giulia Storace, Matteo Trezzi

**Affiliations:** ^1^Divisione di Nefrologia – Dialisi –Trapianto, Ospedale Sant’Andrea, La Spezia, Italy; ^2^Divisione di Chirurgia Vascolare e Trapianto di rene, Azienda Ospedaliera Universitaria San Martino, Genova, Italy; ^3^Servizio di Anatomia Patologica, Azienda Ospedaliera Universitaria San Martino, Genova, Italy; ^4^Clinica Nefrologica, Azienda Ospedaliera Universitaria San Martino, Genova, Italy; ^5^Estor Spa, Pero, Milan, Italy

**Keywords:** Thrombotic miocroangiopathy, Kidney transplantation, Leiden factor V, Protrombin gene

## Abstract

**Introduction:** Thrombotic microangiopathy (TMA) is a serious complication of renal transplantation and is mostly related to the prothrombotic effect of calcineurin inhibitors (CNIs). A subset of TMA (29%-38%) is localized only to the graft.

**Case 1:** A young woman suffering from autosomal dominant polycystic kidney disease (ADPKD) underwent kidney transplant. After 2 months, she showed slow renal deterioration (serum creatinine from 1.9 to 3.1 mg/dl), without hematological signs of hemolytic-uremic syndrome (HUS); only LDH enzyme transient increase was detected. Renal biopsy showed TMA: temporary withdraw of tacrolimus and plasmapheresis was performed. The renal function recovered (serum creatinine 1.9 mg/dl). From screening for thrombophilia, we found a mutation of the Leiden factor V gene.

**Case 2:** A man affected by ADPKD underwent kidney transplantation, with delay graft function; first biopsy showed acute tubular necrosis, but a second biopsy revealed TMA, while no altered hematological parameters of HUS was detected. We observed only a slight increase of lactate dehydrogenase (LDH) levels. The tacrolimus was halved and plasmapheresis was performed: LDH levels normalized within 10 days and renal function improved (serum creatinine from 9 to 2.9 mg/dl). We found a mutation of the prothrombin gene. Only a renal biopsy clarifies the diagnosis of TMA, but it is necessary to pay attention to light increasing level of LDH.

**Conclusion:** Prothrombotic effect of CNIs and mTOR inhibitor, mutation of genes encoding factor H or I, anticardiolipin antibodies, vascular rejection, cytomegalovirus infection are proposed to trigger TMA; we detected mutations of factor II and Leiden factor V, as facilitating conditions for TMA in patients affected by ADPKD.

**Implication for health policy/practice/research/medical education:**

Thrombotic microangiopathy (TMA) is a serious complication of renal transplantation and is mostly related to the prothrombotic effect of calcineurin inhibitors (CNIs). A subset of TMA (29%-38%) is localized only to the graft. Anomalies of coagulation can be permissive for the development of TMA.

## Introduction

Thrombotic microangiopathy (TMA) is a pathological process of microvascular thrombosis, thrombocitopenia and microangiopathic hemolytic anemia, with ischemia and occasionally infarction, affecting particularly the kidney and the brain (1). TMA is a recognized and destructive process in renal transplantation, affecting 3% to 14% of patients on calcineurin inhibitors (CNIs) therapy (2-5). It produces narrowing or occlusion of capillaries, fibrinoid change in the intima of small arteries and subendothelial accumulation of amorphous material in glomeruli. A subset of TMA (29%-38%) is localized only to the graft and patients do not show the classical signs of hemolytic uremic syndrome.

The clinical presentation of TMA is variable, often shows systemic signs of hemolytic uremic syndrome (HUS), with findings of hemolytic-anemia, rapid deterioration of renal function, peripheral schistocytes, and thrombocytopenia, but cases of TMA only localized to the allograft do not present systemic manifestation of HUS.

CNIs, mTOR inhibitors, viral infections, ischemia–reperfusion injury, acute rejection, mutation of genes encoding factor H or I, anticardiolipin antibodies predispose to post-transplant TMA (6,7), but no coagulation genetic alterations were described to facilitate this serious complication of renal transplantation.

We describe two cases of de novo TMA localized only to the graft in autosomal dominant polycystic kidney disease (ADPKD) with thrombophilia.

## Case I

**Figure 1:**
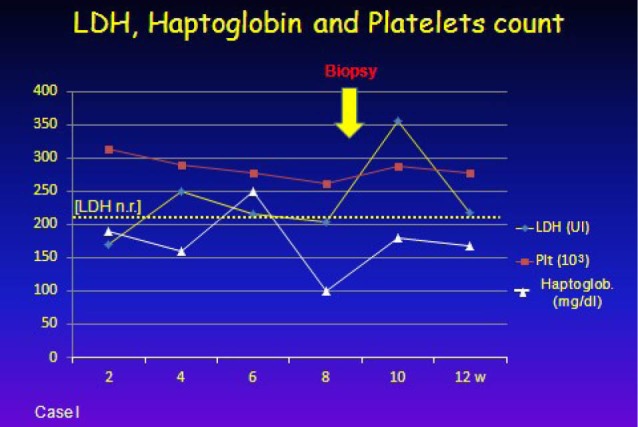
Case I. Lactate dehydrogenase enzyme (LDH) value, haptoglobin level and platelets count before and after biopsy, that showed localized thrombotic microangiopathy after two months from kidney transplant.

**Figure 2:**
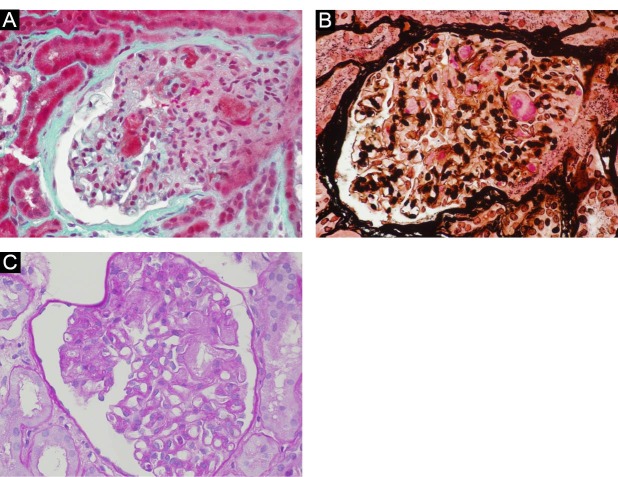
Case I. Renal biopsy specimen demonstrated histopathological glomerular characteristics of TMA (400x) . A) Dilatation of capillaries, stasis and thrombosis( Masson’s trichrome). B) Thrombosis in dilated capillary lumina (Periodic Schiff-Methenamine). C) Mesangiolysis and dilated capillary lumina (Periodic Acid Schiff).

A 39-year-old-woman, suffering from ADPKD, diagnosed at 14-year-old owing to gross hematuria, was on hemodialysis for one year before the transplant. The patient received a kidney transplantation from a 52 years-old deceased due to cerebral hemorrhage with 14 hours of cold ischemic time. The HLA mismatch was 1 and his immunosuppression consisted of two intravenous doses of basiliximab, the first dose (20 mg) preoperatively and the second dose (20 mg) on postoperative day 4, a methyprednisolon taper to 5 mg daily, mycophenolate and tacrolimus as maintenance. We observed an incomplete recovery of renal function (serum creatinine: 1.9 mg/dl). After 2 months from kidney transplantation, she showed a slow renal deterioration (until serum creatinine; 3.9 mg/dl), without humoral signs of hemolytic syndrome. However only a transient increase of lactate dehydrogenase enzyme (LDH) was seen (Figure 1). An allograft kidney biopsy was performed and renal biopsy specimen showed fragmented red blood cells in the glomeruli, partial occlusion of capillaries, fibrinoid change in the intima of small arteries and subendothelial accumulation of amorphous material (Figure 2A-C). C4d was negative. Immediately, temporary withdraw of tacrolimus was performed and a plasmapheresis cycle (five sessions of plasma-exchanges) was made. The renal function slowly recovered and it was stabilized (serum creatinine: 1.8 mg/dl) after two years from the de novo TMA. Genetic coagulation abnormality study was performed and we found a heterozygous mutation of factor V Leiden gene. Search for mutations of gene encoding factor H and I was negative.

## Case II

**Figure 3:**
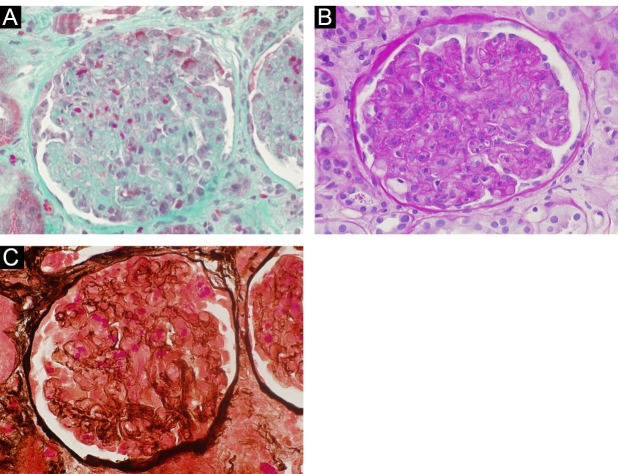
Case II. Renal biopsy specimen showed glomerular lesions in thrombotic microangiopathy (TMA) (400x) . A) Focal fibrinoid necrosis. (Masson’s trichrome). B) Most capillary lumina closed (PAS). C) Thickening of the capillary walls and thrombosis (PASM).

In a 68-year-old man, ADPKD was diagnosed at 57-year-old. In 2012, he underwent kidney transplantation from a 60-year-old woman deceased due cerebral hemorrhage, with 15 hours of cold ischemic time. The HLA mismatch was 4 and the immunosuppression consisted of two intravenous doses of basiliximab (20 mg), a methylprednisolon taper to 5 mg daily, mycophenolate and tacrolimus as maintenance. A prolonged delay graft function with oligo-anuria characterized the clinical course. A first biopsy after 10 days showed an acute tubular necrosis. A second biopsy does not contain glomeruli, and a third biopsy, done at day 45 showed an extensive TMA. The renal biopsy specimen demonstrated arteriolar thrombi, occlusion of glomerular capillaries by amorphous material, fragmented red blood cells in glomeruli (Figure 3A-C). No signs of hemolytic uremic syndrome were detected. Again, we observed only a persistent slight increase of LDH. The tacrolimus was interrupted and LDH levels, in addition to renal function, were strictly observed. LDH level normalized within 10 days, diuresis increased and became effective with partial, but satisfactory recovery of renal function (serum creatinine; 2.9 mg/dl). After four years from the de novo TMA, we observed further amelioration (serum creatinine: 1.5 mg/dl). Also for this patient, genetic coagulation abnormality study was performed, and we found a heterozygous mutation of the prothrombin gene (Factor II).

## Discussion

TMA is a serious complication of renal transplantation, affecting 3% to 14% of patients. It can occur as a recurrent or de novo disease. In a retrospective review by Schwimmer et al (2) between 21 of 742 kidney transplant recipients suffering from TMA, 38% showed localized TMA. Patients with systemic and localized TMA did not differ significantly with respect to age, sex, race, type of donor and pre-transplant diagnosis, and despite systemic and localized TMA have different characteristics and clinical course, long-term graft survival was similarly poor (graft loss 14% to 57% in published case series). Conversely, in other published studies (8,9), a high proportion of patients suffering from localized TMA showed lower rates of TMA-related graft loss (0% to 12%).

We have described two cases of kidney transplantation suffering from TMA due CNIs, facilitated by genetic coagulation abnormalities, with a favorable outcome and a very good follow-up.

Toxicity by CNIs is the main etiologic factor of the disease, because CNIs induce arteriolar vasoconstriction due to production of vasoconstrictive factors, for increased sensitivity to endothelin-I and low synthesis of prostacyclin or nitric oxide. Furthermore, CNIs could promote a pro-coagulant state by enhancing platelet aggregation (10).

Moreover, mTOR inhibitor, viral infection, ischemia-reperfusion injury, viral infection (CMV, Parvovirus B19 and Polioma BK) antibody-mediated rejection and antiphospholipid antibodies are other frequent etiologic agents.

The coagulation abnormality is not investigated enough in this disease, although microvascular thrombosis is the hallmark of TMA.

We have described two different anomalies of coagulation state in APKD patients. The mutation of factor V Leiden (three heterozygous, and one homozygous) was described by Raife et al (11) in a white TMA patient, who had normal von Willebrand factor-cleaving protease (VWCP). On the other hand, no factor II allele abnormality was detected in patients suffering from TMA with normal or deficient VWCP.

Inactivation of factor Va by activated protein C is impaired in patients with factor Leiden mutation. It is possible that, it is implicated in the thrombomodulin/protein C anticoagulant pathway in TMA.

In conclusion, factor V Leiden may be among a constellation of genetic risks permissive of the development of TMA, when CNIs exposition is present, as in the cases we have described here.

Localized TMA has a variable clinical presentation, as no signs of hemolytic anemia, schistocytes, thrombocytopenia and low haptoglobin level was observed. Only a moderate increase of lactate dehydrogenase enzyme was seen and only renal biopsy is really diagnostic.

## Conclusion

TMA is a complication of renal transplantation and is mostly related to the prothrombotic effect of CNI. A subset of TMA (29%-38%) is localized only to the graft, and only slight elevation of LDH level can be seen. Anomalies of coagulation in patients suffering from ADPKD can be permissive for the development of TMA.

## Ethical considerations

Ethical issues (including plagiarism, misconduct, data fabrication, falsification, double publication or submission, redundancy) have been completely observed by the authors.

## Conflicts of interest

The authors declared no competing interests.

## Funding/Support

None.
